# Improving the Photocatalytic Activity of Mesoporous Titania Films through the Formation of WS_2_/TiO_2_ Nano-Heterostructures

**DOI:** 10.3390/nano12071074

**Published:** 2022-03-25

**Authors:** Junkai Ren, Luigi Stagi, Luca Malfatti, Valentina Paolucci, Carlo Cantalini, Sebastiano Garroni, Marzia Mureddu, Plinio Innocenzi

**Affiliations:** 1 Laboratory of Materials Science and Nanotechnology (LMNT), Department of Biomedical Sciences, CR-INSTM, University of Sassari, 07100 Sassari, Italy; j.ren@studenti.uniss.it (J.R.); lstagi@uniss.it (L.S.); lucamalfatti@uniss.it (L.M.); 2 Department of Industrial and Information Engineering and Economy, University of L’Aquila, 67100 L’Aquila, Italy; valentina.paolucci2@univaq.it (V.P.); carlo.cantalini@univaq.it (C.C.); 3 Department of Chemistry and Geology, University of Sassari, 07100 Sassari, Italy; sgarroni@uniss.it (S.G.); m.mureddu6@studenti.uniss.it (M.M.)

**Keywords:** tungsten disulfide, titania, heterostructure, photocatalysis, mesoporous films

## Abstract

Heterostructures formed by anatase nanotitania and bidimensional semiconducting materials are expected to become the next-generation photocatalytic materials with an extended operating range and higher performances. The capability of fabricating optically transparent photocatalytic thin films is also a highly demanded technological issue, and increasing the performances of such devices would significantly impact several applications, from self-cleaning surfaces to photovoltaic systems. To improve the performances of such devices, WS_2_/TiO_2_ heterostructures obtained by incorporating two-dimensional transition metal dichalcogenides layers into titania mesoporous ordered thin films have been fabricated. The self-assembly process has been carefully controlled to avoid disruption of the order during film fabrication. WS_2_ nanosheets of different sizes have been exfoliated by sonication and incorporated in the mesoporous films via one-pot processing. The WS_2_ nanosheets result as well-dispersed within the titania anatase mesoporous film that retains a mesoporous ordered structure. An enhanced photocatalytic response due to an interparticle electron transfer effect has been observed. The structural characterization of the heterostructure has revealed a tight interplay between the matrix and nanosheets rather than a simple additive co-catalyst effect.

## 1. Introduction

Since the discovery of graphene, the attention of researchers has focused on obtaining other two-dimensional (2D) materials by exfoliation of the parent layered bulk materials or via bottom-up routes [1,2]. Stacks of weakly bonded and atomic thick sheets form the layered materials, and different methods have been developed to exfoliate them into single- or few-layer components. Transition metal dichalcogenides (TMDCs), such as molybdenum disulfide (MoS_2_) and tungsten disulfide (WS_2_), are among the most studied layered materials whose functional properties enable applications in electronics and photonics [3]. Among TMDCs, WS_2_ has attracted particular attention because of its outstanding electronic properties, making it an ideal material for the rational design of photocatalysts and photoelectrodes [4,5]. In fact, the WS_2_ bandgap undergoes an indirect (~1.4 eV) to direct (~2.1 eV) transition when the material size is reduced from bulk to monolayer, as an effect of the quantum confinement. WS_2_ nanosheets have been combined with other semiconductors, such as g-C_3_N_4_ [6,7], CdS [8,9], and TiO_2_ [10,11,12], to fabricate heterostructures with enhanced photocatalytic activity for pollutant degradation or water splitting. Although pure TiO_2_ is still considered a standard for photocatalysis, the fast hole-charge recombination rate represents an intrinsic limit [13]. For this reason, the development of titania heterostructures with chalcogenide materials is the subject of intensive investigation in the quest for an efficient method to produce energy from sunlight.

Up to now, nanocrystalline anatase titania (TiO_2_) in the form of micro- or nano-particles has represented the first choice for photocatalytic application. The low production cost, the exceptional stability under irradiation, and the ease of synthesis make the nanotitania an excellent photocatalytic material. A few articles have reported the formation of heterostructures based on nanocrystalline titania particles (both dense and mesoporous) sensitized with WS_2_ [12,14]. In all cases, the formation of WS_2_ occurred in situ on the TiO_2_ particle surface via photochemical reduction of (NH_4_)_2_WS_4_. The formation of heterostructures, obtained by coupling two semiconductors with different energy levels, besides enhancing the photocatalytic performances under UV-light irradiation [15], could also extend the range of activity to the visible light.

More recently, however, the use of TiO_2_ powders and nanoparticles has been severely limited due to the classification as category 2 “suspected carcinogen by inhalation”, made by the Committee for Risk Assessment of the European Chemical Agency [16]. Therefore, the synthesis of TiO_2_ mesoporous films appears as an attractive alternative. The material in the form of a thin film does not pose inhalation health risks and provides an efficient platform for photocatalysis due to the high surface area. However, the fabrication of optically active heterostructures by integrating 2D layers into mesoporous ordered titania films is still a challenging target to achieve. To the best of our knowledge, there is only one published report about the synthesis of a porous titania coating supporting WS_2_ [17]. These heterostructures, however, were prepared by forming a thin layer of 2D nanosheets directly on the porous surface. This synthesis method creates a sharp separation between the titania porous film and the TMDC phase.

In our previous works, we have successfully introduced graphene and boron nitride sheets into titania mesoporous films via evaporation-induced self-assembly [18,19]. This process allows direct incorporation of mechanically exfoliated 2D materials in the precursor sol. The method requires integrating the self-assembly process with the 2D materials without disrupting the self-organization during the film processing. The final nanocrystalline mesoporous matrix is obtained by thermal annealing. After the processing, the 2D materials are homogeneously dispersed within the matrix while preserving the high surface area provided by the mesoporous structure. The fabrication of a robust TiO_2_-based heterostructure in the shape of thin mesoporous ordered films is expected to improve the functional properties thanks to the higher surface area and better diffusivity in the pore structure.

TiO_2_-WS_2_ heterostructures have been fabricated and tested for photocatalysis in the present work. Integration of photoactive heterostructures into functional devices should have a significant impact on improving the photocatalysis performance.

## 2. Results and Discussion

**Scheme 1:**
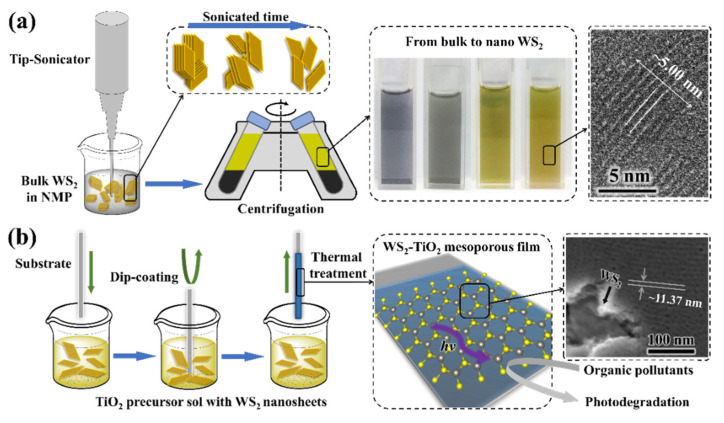
Schematic of (**a**) the exfoliation of 2D WS_2_ nanosheets via tip-sonication, and (**b**) the formation of WS_2_-TiO_2_ mesoporous film heterostructures via dip-coating.

A critical step in the synthesis of TiO_2_-2D WS_2_ heterostructures is the production of WS_2_ sheets of controlled properties, employing a feasible and reproducible fabrication method. The exfoliation of 2D WS_2_ has been achieved by sonication of crystalline WS_2_ powders (WS_2_-P) in 1-methyl-2-pyrrolidinone (NMP) using a sonicator tip (Scheme 1a). Centrifugation at different rotational speeds has allowed precipitating the unexfoliated aggregates (WS_2_-U), and further separating the exfoliated WS_2_ into large and small nanosheets (WS_2_-L and WS_2_-S), respectively. With the reduction of size and dimension, the color of WS_2_ dispersions gradually changes from dark grey to brown and yellow. The TiO_2_ mesoporous films have been prepared via a template-assisted self-assembly route. The WS_2_-TiO_2_ heterostructures form by incorporating the WS_2_ nanosheets into TiO_2_ films in the precursor sol and deposition of thin films by dip-coating (Scheme 1b). During the solvent evaporation, the templating micelles self-assemble into an ordered array while the WS_2_ sheets disperse within the matrix without disrupting the self-assembly.

### 2.1. Exfoliation of WS_2_ Nanosheets

A set of complementary analyses has been used to characterize the size and structure of the WS_2_ samples after sonication and centrifugation.

**Figure 1:**
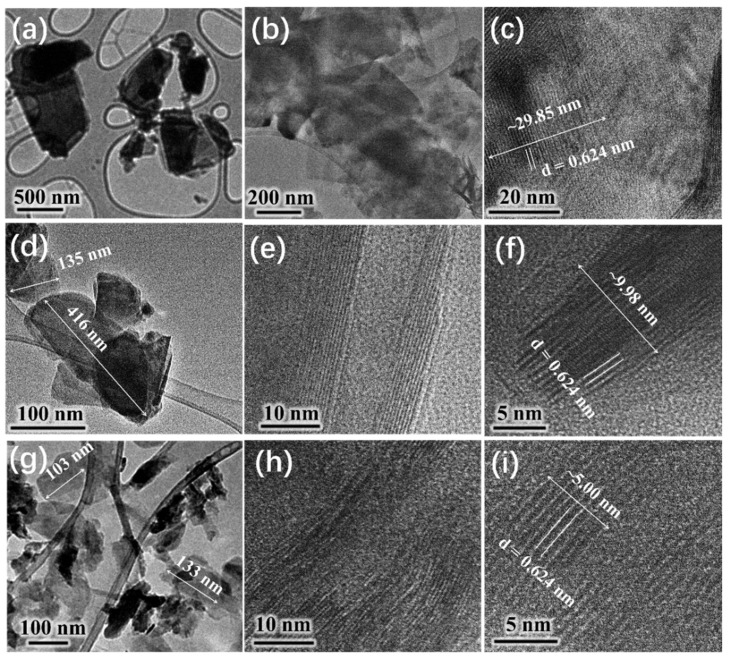
TEM images of the WS_2_ products after sonication. (**a**–**c**) WS_2_-U, (**d**–**f**) WS_2_-L, and (**g**–**i**) WS_2_-S.

The transmission electron microscopy (TEM) images in Figure 1 confirm that higher centrifugation rates allow for the collection of thinner and smaller nanosheets in the supernatants. The lateral size of unexfoliated WS_2_ (WS_2_-U) is over 1 μm, and the thickness is in the range of tens of nanometers, suggesting that WS_2_-U still shows the bulk crystal characteristics. On the contrary, the exfoliated WS_2_ nanosheets have a lateral size in the range of 100–400 nm. The folding edges of WS_2_ platelets reported in Figure 1c,f,i enable direct observation of the layered structure through the interplanar distance of the (002) planes. The interlayer d-spacing is ~0.624 nm, which is in good agreement with the value reported for the 2H-WS_2_ structure [20]. Interestingly, the thicknesses of WS_2_-L (~10 nm) and WS_2_-S (~5 nm) are much thinner than WS_2_-U, corresponding to ~16 and ~8 monoatomic layers, respectively.

**Figure 2:**
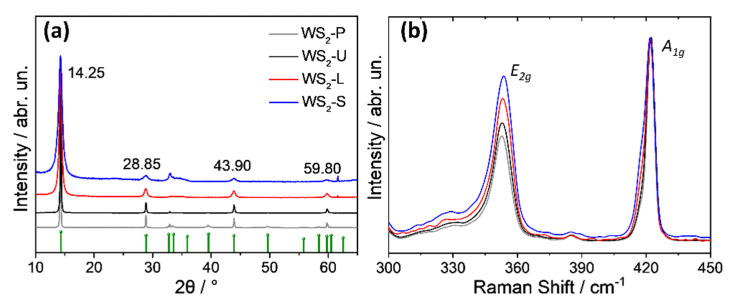
(**a**) XRD patterns and (**b**) Raman spectra of WS_2_-P (grey line), WS_2_-U (black line), WS_2_-L (red line), and WS_2_-S (blue line). The green marks at the bottom of (**a**) are the diffraction peaks of 2H-WS_2_ (PDF Card ICDD No. 00-008-0237).

Figure 2a shows the X-ray diffraction (XRD) patterns of the different WS_2_ layers’ structures. An intense diffraction peak at 14.25° from the characteristic (002) reflection characterizes the 2H-WS_2_ phase, and three weaker diffraction peaks at 28.85°, 43.90°, and 59.80° corresponding to the (004), (006), and (008) planes were also detected [21]. According to Bragg’s equation, the d-spacing of (002) is ~0.621 nm, in good accordance with the TEM results. The full width at half-maximum (FWHM) of all XRD peaks shows a widening trend when the dimension of WS_2_ reduces from the bulk to nanoscale, in agreement with the Scherrer law.

Raman analysis in Figure 2b supports the XRD and TEM results. The bands peaking at ~422.0 and 352.5 cm^−1^ are assigned to the first-order *A*_1*g*_ and *E*_2*g*_ Raman modes, which originate from the out-of-plane and in-plane vibrations, respectively [22]. A detailed analysis of the Raman bands (Supplementary Figure S1) confirms the size reduction of WS_2_ as a function of the sonication treatment. For example, by comparing WS_2_-P with WS_2_-S, it can be found that the frequency difference of *A*_1*g*_ and *E*_2*g*_ changed from 69.5 to 68.5 cm^−1^, the FWHM of the *A*_1*g*_ mode widened from 4.32 to 6.18 cm^−1^, and the relative intensity of *A*_1*g*_/*E*_2*g*_ decreased from 1.92 to 1.23.

**Figure 3:**
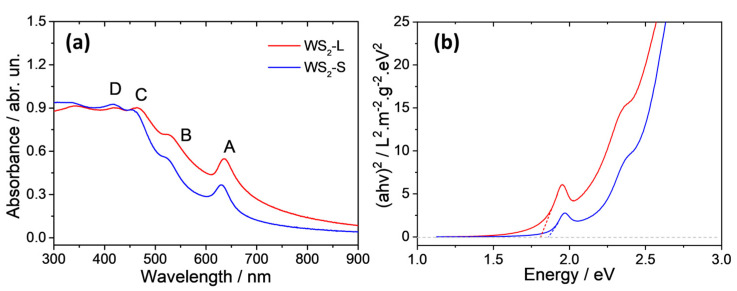
(**a**) UV-Vis absorption spectra and (**b**) Tauc plots of WS_2_ nanosheets (WS_2_-L red line, WS_2_-S blue line).

Figure 3a shows the UV-Vis absorption spectra of WS_2_ dispersions in EtOH. No obvious absorption bands were detected from the bulk WS_2_ samples (WS_2_-P and WS_2_-U) because their indirect bandgap can reach ~1.4 eV [23], which is in the near-infrared region (see Supplementary Figure S2). When the WS_2_ size is reduced to the nanoscale, four bands can be distinctly observed (labeled as A, B, C, and D). In the case of WS_2_-L, the A and B excitons at 635 and 530 nm originate from the direct gap transitions at the K point in the Brillouin zone, while the C and D bands at 464 and 418 nm are due to the direct transitions from the deep valence to the conduction band [24,25]. In WS_2_-S, these absorption bands show a hypsochromic shift to 629, 523, 456, and 415 nm, respectively, attributed to the quantum confinement in nanosheets [26].

The band A at 635 nm corresponds to the lowest optical bandgap, which can be evaluated using the Tauc equation (see Figure 3b) [27]:

(αhν)^n^ = A(hν − E_g_) (1)

where hν is the photon energy, α is the energy absorption coefficient (calculated from UV-Vis absorption spectra), A is the absorption edge width parameter, E_g_ is the bandgap, and the exponent n depends on the type of optical transition in the gap region (*n* equals 2 for a direct transition). WS_2_-L and WS_2_-S have a calculated bandgap of 1.82 and 1.88 eV, respectively. Both these values are higher than the corresponding bulk value of ~1.4 eV and close to the bandgap of ~2.1 eV reported in the literature for a single layer [22]. These results suggest that a reduction of the number of layers can lead to a crossover transition from an indirect bandgap in the bulk to a direct bandgap in the monolayer.

### 2.2. Construction of WS_2_-TiO_2_ Heterostructures

The structural and optical properties of mesoporous thin films have been extensively investigated to understand how the insertion of WS_2_ nanosheets can affect the heterostructure formation. Bulk WS_2_ and their nanosheets (both large and small) have been incorporated into the titania mesoporous matrices to form namely TiO_2_-WS2 (U), TiO_2_-WS_2_ (L), and TiO_2_-WS_2_ (S) heterostructures, while the undoped TiO_2_ was used as a reference film for comparison.

**Figure 4:**
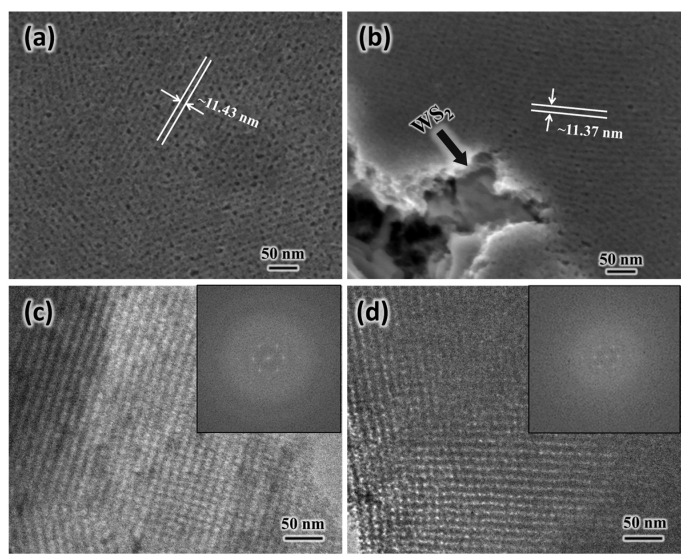
(**a**,**b**) SEM and (**c**,**d**) TEM images of (**a**,**c**) undoped mesostructured TiO_2_ and (**b**,**d**) WS_2_-doped mesostructured TiO_2_ films. The insets in (**c**,**d**) show the corresponding FFT patterns.

Figure 4a,b shows the field emission scanning electron microscope (FE-SEM) images of the mesostructured titania films after annealing. The image at high magnification indicates that thermal removal of the template leaves a well-organized mesoporous structure. The surface plot analysis provided a wall-to-wall average distance of 11.4 nm for both pure mesostructured TiO_2_ and its heterojunction. The TEM images in Figure 4c,d allow more precise observation of the ordered pore arrangement. The morphologies are compatible with a body-centered cubic structure with an Im-3m symmetry [19,28]. According to the Fast Fourier Transform (FFT) patterns, the cell parameter is ~11.8 nm (11.74 nm for undoped TiO_2_, 11.93 nm for its heterostructure). The ordered mesoporosity within the nanocomposite films indicates that the presence of WS_2_ sheets does not disrupt the self-assembly process.

**Figure 5:**
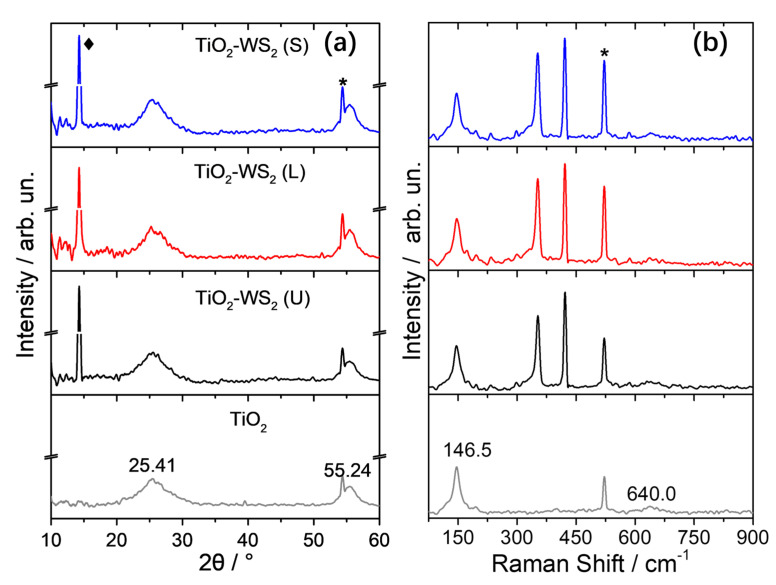
(**a**) XRD patterns and (**b**) Raman spectra of WS_2_-TiO_2_ films: undoped TiO_2_ (grey line), TiO_2_-WS_2_-U (black line), TiO_2_-WS_2_-L (red line), and TiO_2_-WS_2_-S (blue line). The signal at 521.5 cm^−1^, marked with an asterisk, is caused by the monocrystalline Si wafer.

XRD patterns and Raman spectra have been collected to determine the crystal structure of TiO_2_-WS_2_ films deposited on silicon wafer substrates. The two sharp diffraction peaks at 14.25° and 54.36° (Figure 5a) are assigned to the characteristic (002) reflection in the 2H-WS_2_ phase (marked by a rhombus ◆) and the (311) plane of the Si wafer surface (marked by an asterisk *). The signals at 25.41° and 55.24° are attributed to (101) and (211) reflections of the anatase phase, obtained from amorphous titania after the annealing process [10,29]. By applying the Scherrer equation to the (101) reflection, we have estimated the anatase crystallite size as 1.78, 1.85, 2.20, and 2.31 nm for TiO_2_, TiO_2_-WS_2_ (U), TiO_2_-WS_2_ (L), and TiO_2_-WS_2_ (S), respectively. Despite only minor differences among XRD patterns, the data suggest that the 2D structures promote anatase crystallization via heterogeneous nucleation. Interestingly, this effect is emphasized with the decrease of the WS_2_ size, because of the higher specific surface area.

Figure 5b allows comparing the Raman spectra of the different TiO_2_-WS_2_ heterostructures. The Raman band at 146.5 cm^−1^ and the weak signal at 640.0 cm^−1^ are assigned to the *E_g_* vibration mode of O-Ti-O in the anatase phase [30]. These bands show the same intensity and position in all four spectra. On the contrary, the two sharp bands at ~422.0 and 352.5 cm^−1^, attributed to the first-order *A*_1*g*_ and *E*_2*g*_ modes of WS_2_, are only present in WS_2_-TiO_2_ films, suggesting the successful incorporation of WS_2_ platelets into the titania layers. Interestingly, no signals were detected from the O-W-O stretching in the range of 750–900 cm^−1^ [31], indicating that the N_2_ flow can effectively avoid the oxidation of WS_2_ samples during the thermal annealing of the titania films.

Spectroscopic ellipsometry has also been used to estimate the thickness and refractive index of the samples. The films had a similar thickness in the range of 130–140 nm with a general experimental error of ~10 nm (Supplementary Figure S3a), confirming that the incorporation of WS_2_ platelets does not cause shrinkage or expansion of TiO_2_ thin films. The refractive index of the samples in the 380–900 nm range constantly increased by decreasing the WS_2_ nanosheet size (Supplementary Figure S3b), similarly to what was observed for the crystal size of TiO_2_ anatase.

**Figure 6:**
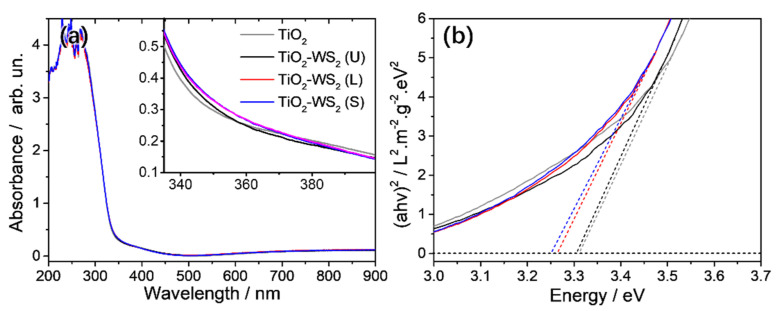
(**a**) UV-Vis absorption spectra and (**b**) Tauc plots of WS_2_-TiO_2_ films: undoped TiO_2_ (grey line), TiO_2_-WS_2_-U (black line), TiO_2_-WS_2_-L (red line), and TiO_2_-WS_2_-S (blue line). The inset in (**a**) shows an enlarged region in the 330–400 nm region.

Figure 6a shows the UV-Vis absorption spectra of the mesoporous films deposited on silica glass substrates. There were no evident differences among these samples, however a closer look at the ultraviolet range revealed that TiO_2_-WS_2_ (L) and (S) films have a slightly higher absorption (around 0.025 in intensity) than TiO_2_ and TiO_2_-WS_2_ (U). The higher absorption is caused by the incorporation of WS_2_ nanosheets (see Figure 3a) which, actually, does not allow observing the A–D bands of layered WS_2_ from the UV-Vis spectra of TiO_2_-WS_2_ films, because of a limited doping amount. The optical transmittance of the four samples (Supplementary Figure S4) allowed for plotting the Tauc curves in Figure 6b, which shows the effect of 2D WS_2_ on the bandgap of the films. The bandgap value shifted from 3.31 eV (undoped TiO_2_) to 3.30, 3.27, and 3.25 eV, respectively, for the three WS_2_-TiO_2_ films. In accordance with the previous results, smaller-sized WS_2_ sheets contributed to a larger bathochromic shift of the optical gap.

### 2.3. Evaluation of Photocatalytic Activity in WS_2_-TiO_2_ Heterostructures

**Figure 7:**
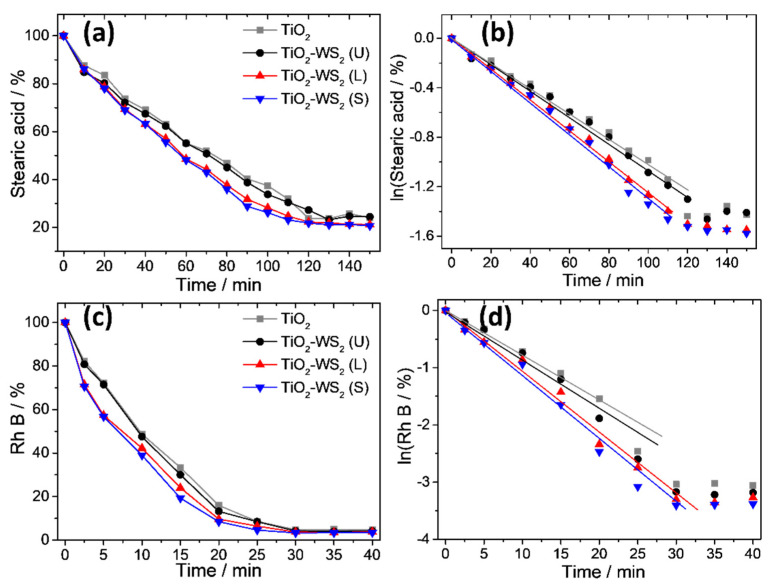
Photoinduced degradation of (**a**) stearic acid and (**c**) RhB on the WS_2_-TiO_2_ films, and the pseudo-first-order fit of (**b**) stearic acid and (**d**) RhB decay at increasing times of UV irradiation: undoped TiO_2_ (grey line), TiO_2_-WS_2_-U (black line), TiO_2_-WS_2_-L (red line), and TiO_2_-WS_2_-S (blue line).

Stearic acid has been used to measure the photocatalytic response of the heterostructures (Figures S5 and S6) by monitoring the absorption intensity of the -CH_3_ and -CH_2_ vibrational modes in the 2945–2845 cm^−1^ range using FTIR spectroscopy (see Supplementary Figure S7). The degradation rate of stearic acid (stearic acid/%) has been determined by the following equation:

stearic acid/% = I_t_/I_0_ * 100% (2)

where I_t_ stands for the absorption maximum intensity as a function of irradiation time and I_0_ is the initial value of the absorption maximum intensity before exposure (at t = 0). Figure 7a shows the photoinduced degradation curves of stearic acid cast on the different mesoporous films. After around 2 h of UV exposition, ~80% of the stearic acid can be degraded on the four samples. The photodegradation data follow the pseudo-first-order kinetics according to Figure 7b. Therefore, the degradation curves have been fitted by an exponential decay law:

I(t) = I_0_ * e^−kt^ (3)

where the parameter k is the degradation rate. The k value for undoped TiO_2_ film was calculated to be ~0.0105 min^−1^, and the k for the TiO_2_-WS_2_ (U) film was similar, ~0.0107 min^−^^1^, which suggests that the bulk WS_2_ does not change the photoactivity of titania. Interestingly, the k values of TiO_2_-WS_2_ (L) and (S) films (~0.0118 and 0.0121 min^−1^) showed ≈20% enhancement with respect to bare TiO_2_ or TiO_2_-WS_2_ (U).

The photocatalytic activity of the heterostructures has also been evaluated using Rhodamine B (RhB) as a probe dye by calculating the absorption intensity in the 450–600 nm region (see Supplementary Figure S8). Figure 7c shows that ~95% of the RhB can be degraded on the four films after the UV exposition of only 30 min. According to Figure 7d, the k values of TiO_2_-WS_2_ (L) and (S) films (~0.1063 and 0.1102 min^−1^) exhibited a 35% improvement compared to bare TiO_2_ or TiO_2_-WS_2_ (U) films (~0.0808 and 0.0843 min^−1^). It shows a trend similar to stearic acid even if the photodegradation rate is higher. It must be underlined that photodegradation measured using infrared absorption corresponds to an effective degradation of the molecule. Photodegradation data obtained by UV-Vis, on the other hand, are related to a decrease in the optical absorption/emission of the probe dye. The change in the absorption/emission does not necessarily indicate a full degradation and removal of the molecule.

The experiment of photocatalysis has been reproduced three times with the same samples. The small standard deviation (Supplementary Figure S6) indicates the good reproducibility of the photocatalysis performance and the photostability of the heterostructures. Some recent works about the photodegradation properties of TiO_2_ composites are summarized for comparison in Supplementary Table S1. Most of these publications focus on the photodegradation of dyes in aqueous solutions, such as methylene blue and RhB, by recording UV-Vis changes [32,33,34,35,36,37,38]. Actually, the full degradation of organic pollutants cannot be truly detected from the UV-Vis changes, but instead from the infrared vibrations [18,19]. On the other hand, measures performed in a liquid are far from a practical case where surfaces are required for applications. It should be underlined that a 20–35% increase in terms of photocatalytic performances in an optically transparent thin film represents a significant technological improvement.

Previous works have reported an intrinsic photocatalytic activity of WS_2_ nanomaterials, which is usually measured in solutions [4,39]. However, the three WS_2_ obtained in this work did not show photocatalytic degradation of stearic acid if deposited and dried on a Si wafer (see Supplementary Figure S5). Therefore, the enhancement of the photocatalytic activity measured on the TiO_2_-WS_2_ samples cannot be merely attributed to an additive effect of the two materials but rather to the formation of a synergistic heterostructure.

**Scheme 2:**
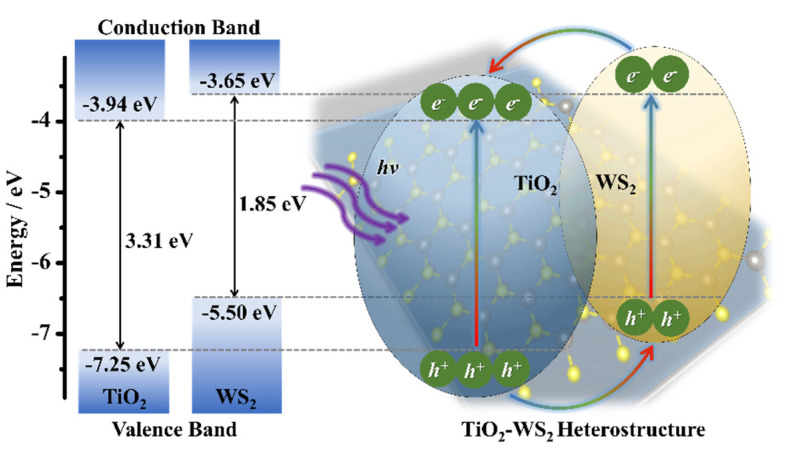
Schematic illustration of the enhanced photocatalysis from the WS_2_-TiO_2_ heterostructure film.

As shown in Scheme 2, the increase in the photocatalytic performances of WS_2_-TiO_2_ heterostructures can be explained considering the interparticle electron transfer (IPET) mechanism. According to the current knowledge, in fact, the valence band (VB) values are around −7.25 and −5.50 eV for TiO_2_ and WS_2_, respectively [40,41]. By coupling these values with the bandgap calculated via Tauc plots, the corresponding conduction bands (CB) can be estimated to be −3.94 and −3.65 eV for TiO_2_ film and WS_2_ nanosheets in our case, respectively. The band offsets allow constructing a plausible band diagram of the heterojunction. The photogenerated electrons migrate efficiently from the CB of WS_2_ to that of TiO_2_, while hole transfer occurs from the VB of TiO_2_ to that of WS_2_ [11,42]. This process results in an efficient charge separation on the hetero-interface of WS_2_-TiO_2_.

## 3. Conclusions

Optically transparent heterostructures formed by the integration of well-dispersed WS_2_ nanosheets into mesoporous ordered titania thin films have been successfully fabricated via self-assembly. The fabrication of the nanocomposite films has been achieved via two separate steps. The first one was the controlled mechanical exfoliation of WS_2_ by tip-sonication to obtain few-layer nanosheets. The second stage was the addition of the WS_2_ layers to the titania precursor sol to form mesoporous nanocrystalline anatase films via self-assembly. As a result, the integration of WS_2_ into the titania matrix did not affect the film thickness or the organized porosity, which appeared monodispersed and well-organized throughout the matrix. In addition, controlled thermal annealing prevented WS_2_ oxidation and promoted anatase formation.

The properties of WS_2_-TiO_2_ heterojunctions were affected by the size of the embedded 2D layers. Smaller-sized WS_2_ sheets had a larger surface area that formed diffused hetero-interfaces within the porous titania structure. At the same time, the bandgap of nanosized WS_2_ sheets blue-shifted towards the direct value of the WS_2_ monolayer, favoring an interparticle electron transfer between the two semiconductors. Due to the fine tailoring of the WS_2_-TiO_2_ heterojunctions, the films showed an enhanced photocatalytic activity (+ 20% or 35%) with respect to the undoped TiO_2_ and the mesoporous films containing unexfoliated WS_2_. The formation of TiO_2_-WS_2_ heterostructures in optically transparent thin films with high photocatalytic activity represents a significant improvement for several technological applications.

## 4. Experimental Section

### 4.1. Chemicals

Crystalline tungsten (IV) sulfide powder (WS_2_, 2 μm, 9%. Aldrich, St. Louis, USA), 1-Methyl-2-pyrrolidinone (NMP, 99%, Sigma-Aldrich, St. Louis, MI, USA), titanium(IV) chloride (TiCl_4_, Aldrich, 99.9%), ethanol (EtOH, Sigma-Aldrich, 99.5%), Pluronic F-127 (∼12,600 g·mol^−1^, Aldrich), stearic acid (Sigma-Aldrich, 97%), and deionized water were used.

### 4.2. Exfoliation of WS_2_ Nanosheets

The WS_2_ nanosheets were prepared by a sonication-assisted liquid-phase exfoliation method. The commercial WS_2_ powders (WS_2_-P, 250 mg) were dispersed into NMP (125 mL) and then sonicated using a probe tip for 5 h (500 W, 40% amplitude). To avoid thermal oxidation of products, the tip-pulse was on for 5 s and off for 2 s, and an ice-water bath was used to cool down the temperature of WS_2_ dispersions.

The nanosheets were collected and separated in accordance with size by centrifuging at different speeds. Firstly, centrifugation at 2000 rpm for 10 min was carried out to precipitate the unexfoliated WS_2_ (WS_2_-U). Secondly, the supernatant was centrifugated at 4000 rpm for 10 min to precipitate the large exfoliated WS_2_ (WS_2_-L). Thirdly, the above supernatant was further centrifugated at 8000 rpm for 10 min to collect the small exfoliated WS_2_ (WS_2_-S). To remove the residual NMP, the products were washed three times by EtOH and dried at 60 °C.

### 4.3. Synthesis of WS_2_-TiO_2_ Film Heterostructures

The mesoporous films were synthesized by an evaporation-induced self-assembly method. At first, Pluronic F-127 (1.3 g) was dissolved in EtOH (46.8 mL), and TiCl_4_ (2.2 mL) was added into the solution. After stirring for 15 min, deionized water (3.6 mL) was dropped into the mixtures. The final molar ratio was TiCl_4_/EtOH/F-127/H_2_O = 1:40:0.005:10. Then, WS_2_ dispersions (500 μL, 5 mg mL^−1^ in EtOH) were added into the precursor sol (10 mL).

Silicon wafer and silica glass were used as the substrates to dip-coat films. The substrates were immersed in the WS_2_ titania sols with a withdrawal rate of 10 cm min^−1^ and kept for 30 s before extraction. The relative humidity (RH) was kept under 30% by a dried airflow. Then, the obtained films were firstly dried at 60 °C in air for 10 h and then thermally annealed at 450 °C for 1 h in a nitrogen atmosphere (see Supplementary Figure S9).

### 4.4. Material Characterizations

Transmission electron microscopy (TEM) images were obtained by an FEI Tecnai 200 microscope working with a field emission electron gun operating at 200 kV.

Scanning electron microscope (SEM) images were captured by a ZEISS GeminiSEM 500 microscope working at an accelerating voltage of 2 kV.

Raman spectra were collected in the 65–1555 cm^−1^ range with a 3–5 cm^−1^ resolution using a Senterra confocal Raman microscope (Bruker, 633 nm laser, 0.2 mW power, and 100× objective).

The X-ray diffraction (XRD) pattern was recorded by a high-resolution diffractometer (Rigaku SmartLab X-ray diffractometer equipped with a rotating anode, 9 kW) with a Cu Kα line (λ = 1.5406 Å) operating at 40 kV and 150 mA.

Ultraviolet-visible (UV-Vis) spectra were obtained by a Nicolet Evolution 300 UV-Vis spectrophotometer (Thermo Fisher, Waltham, MA, USA) with a bandwidth of 1.5 nm.

Spectroscopic ellipsometry (α-Wollam) with fixed-angle geometry was used to measure the thickness and refractive index of the films, which were analyzed via CompleteEASE 4.2 software. A transparent model was used to calculate the refractive index.

### 4.5. Evaluation of Photocatalytic Activity

Stearic acid was selected as the molecular probe to evaluate the photocatalytic activity of the mesoporous WS_2_-TiO_2_ films. The change of vibrational modes in the 2945–2845 cm^−1^ range (-CH_2_ and -CH_3_ stretching) was used to characterize the photodegradation of stearic acid on different films. The process could be quantified by the corresponding integral of the infrared bands as a function of the irradiation time. Herein, Fourier transform infrared (FTIR) spectra were plotted by an infrared Vertex 70 interferometer (Bruker).

At first, stearic acid was dissolved in EtOH (3.3 mg mL^−1^). Then, the solution (100 μL) was deposited on the films by spin-coating at 1500 rpm for 30 s. The films covered by stearic acid were irradiated under 365 nm light from a UV lamp (Spectroline, ENF-280C/FE) at a distance of 0.5 cm. The radiation time was fixed from 0 to 150 min, and FTIR spectra of these samples were recorded immediately after illumination. The photocatalysis testing has been repeated three times to prove the reproducibility of the results.

RhB was also selected as another molecular probe to verify the photocatalytic activity of WS_2_-TiO_2_ films by monitoring the decreasing integral intensity of absorption in the range of 450–600 nm. At first, RhB was dissolved in EtOH (10^−5^ M). Then, the RhB solution (100 μL) was deposited on the films by spin-coating at 1500 rpm for 30 s. The films covered by RhB were irradiated under 365 nm light from a UV lamp at a distance of 10 cm. The radiation time was fixed from 0 to 40 min, and absorption spectra of these samples were recorded immediately after illumination.

## Data Availability

Original data are available upon request to the corresponding author.

## Supplementary Materials

The following supporting information can be downloaded at: https://www.mdpi.com/article/10.3390/nano12071074/s1, Figure S1: Raman spectra of WS_2_ products; Figure S2: UV-Vis spectra of bulk WS_2_; Figure S3: Thickness and refractive index of the composite films; Figure S4: UV-Vs spectra of WS_2_-TiO_2_ films; Figure S5: Reference spectra of photodegradation of stearic acid; Figure S6: Photodegradation of stearic acid and RhB on WS_2_-TiO_2_ films; Figure S7: Photodegradation of stearic acid as a function of UV exposure time; Figure S8: Photodegradation of RhB as a function of UV exposure time; Figure S9: Pictures of the experimental set-up; Table S1: Photodegradation data related to different TiO_2_ photocatalysts. References [18,19,32,33,34,35,36,37,38] are cited in the supplementary materials.
