# Supplementary material for: Improving the Photocatalytic Activity of Mesoporous Titania Films through the Formation of WS2/TiO2 Nano-Heterostructures
Source: Nanomaterials (Basel). 2022 Mar 25;12(7):1074. doi: 10.3390/nano12071074 (PMC9000319; doi:10.3390/nano12071074)
Supplement: Supplementary file 1 [file nanomaterials-12-01074-s001.zip › nanomaterials-1641302-supplementary.pdf]

Supplementary Materials

Improving the Photocatalytic Activity of Mesoporous Titania Films through the Formation of WS_2/TiO_2 Nano-Heterostructures

Junkai Ren [1], Luigi Stagi [1], Luca Malfatti [1], Valentina Paolucci [2], Carlo Cantalini [2], Sebastiano Garroni [3], Marzia Mureddu [3] and Plinio Innocenzi [1,*]

[1] Laboratory of Materials Science and Nanotechnology (LMNT), Department of Biomedical Sciences, CR-INSTM, University of Sassari, Sassari 07100, Italy; j.ren@studenti.uniss.it (J.R.); lstagi@uniss.it (L.S.); lucamalfatti@uniss.it (L.M.)

[2] Department of Industrial and Information Engineering and Economy, University of L'Aquila, L'Aquila 67100 , Italy; valentina.paolucci2@univaq.it (V.P.); carlo.cantalini@univaq.it (C.C.)

[3] Department of Chemistry and Pharmacy, University of Sassari, Sassari 07100, Italy; sgarroni@uniss.it (S.G.); m.mureddu6@studenti.uniss.it (M.M.)

* Correspondence: plinio@uniss.it

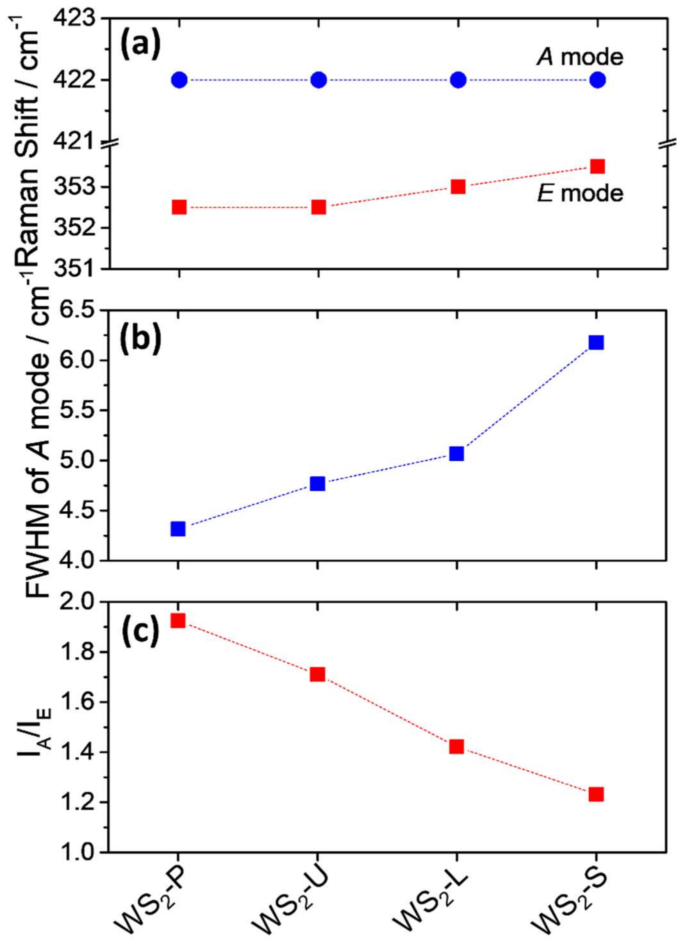

Figure S1. Raman spectroscopy analysis of the WS_2 products, namely WS_2-P, WS_2-U, WS_2-L, and WS_2-S: (a) the frequency shift of A_{1g} and E_{2g} modes, (b) FWHM of A_{1g} modes, and (c) the relative intensity between A_{1g} and E_{2g} modes.

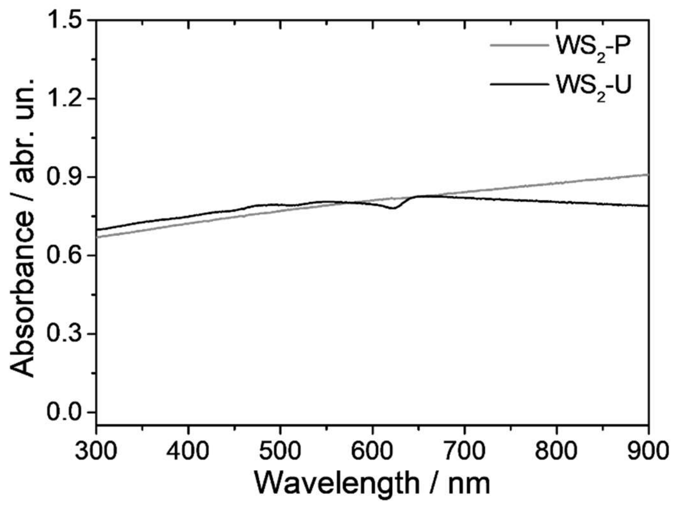

Figure S2. UV-Vis absorption spectra of bulk WS_2 (WS_2-U grey line, WS_2-P black line).

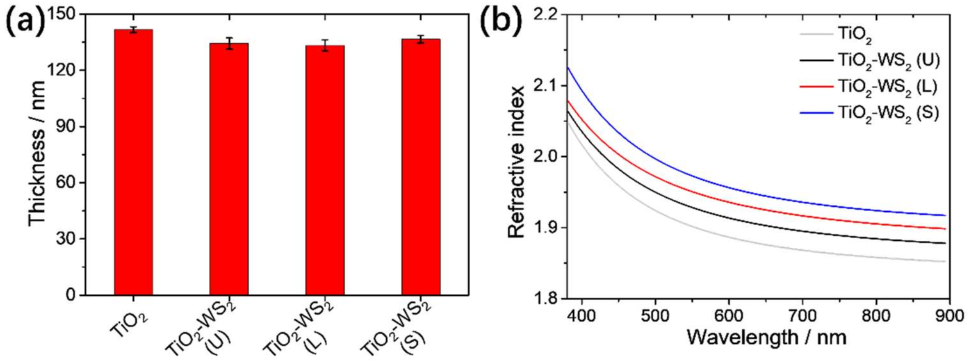

Figure S3. (a) The thickness and (b) refractive index as a function of wavelength of the TiO_2 and WS_2-TiO_2 films according to spectroscopic ellipsometry.

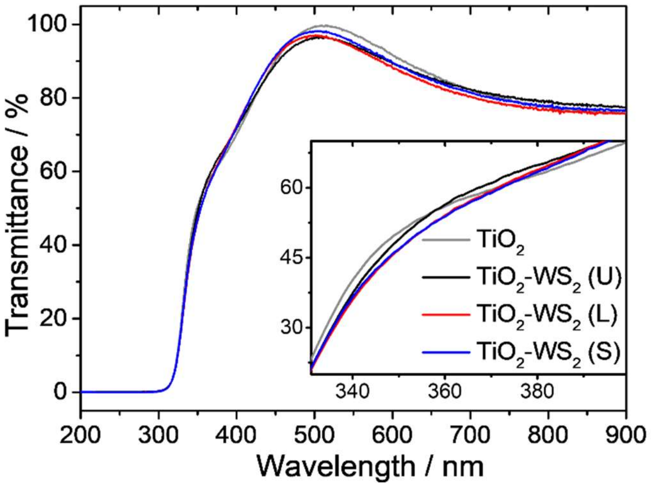

Figure S4. UV-Vis transmittance spectra of WS_2-TiO_2 films: undoped TiO_2 (grey line), TiO_2-WS_2-U (black line), TiO_2-WS_2-L (red line), and TiO_2-WS_2-S (blue line). The inset in (a) shows an enlarged region in 330 – 400 nm.

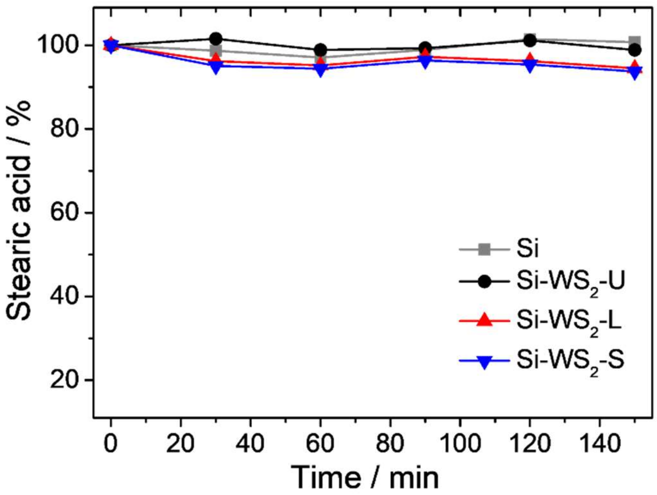

Figure S5. Photodegrading results of stearic acid deposited on bare silicon substrate with/without WS_2 samples: bare silicon (grey line), Si-WS_2-U (black line), Si-WS_2-L (red line), and Si-WS_2-S (blue line).

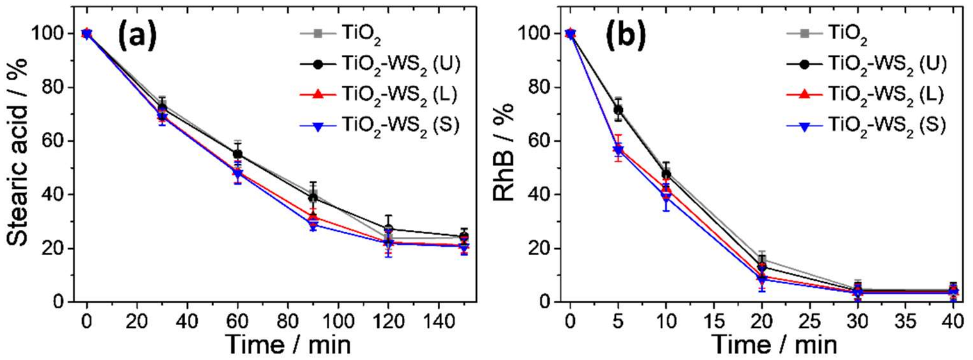

Figure S6. Photoinduced degradation of (a) stearic acid and (b) RhB on the WS_2-TiO_2 films at increasing times of UV irradiation: undoped TiO_2 (grey line), TiO_2-WS_2-U (black line), TiO_2-WS_2-L (red line), and TiO_2-WS_2-S (blue line). Error bars represent the standard deviations of duplicate runs.

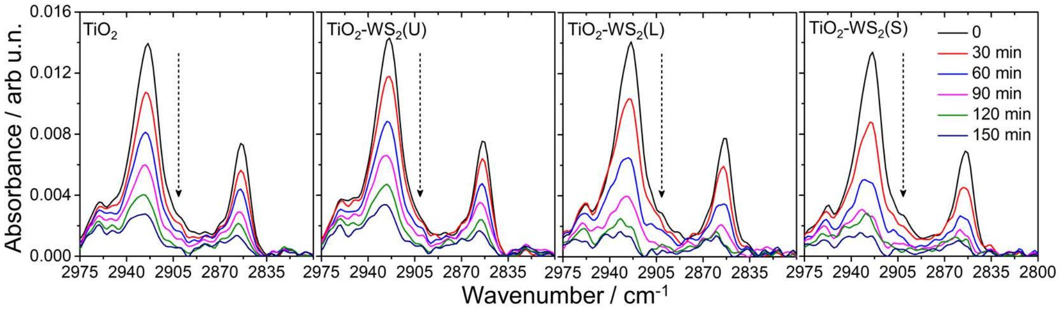

Figure S7. Degradation of stearic acid on different films as a function of UV exposure time (from 0 up to 150 min). The FTIR spectra have been recorded in absorption mode in the range of 2975 – 2800 cm^{-1}.

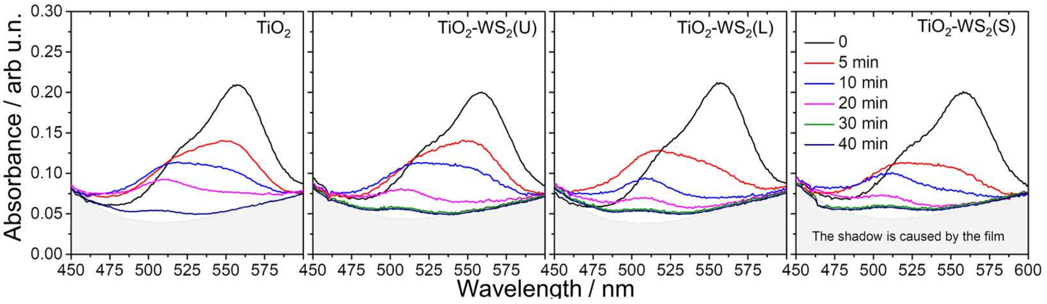

Figure S8. Degradation of RhB on different films as a function of UV exposure time (from 0 up to 40 min). The UV-Vis spectra have been recorded in absorption mode in the range of 450 – 600 nm.

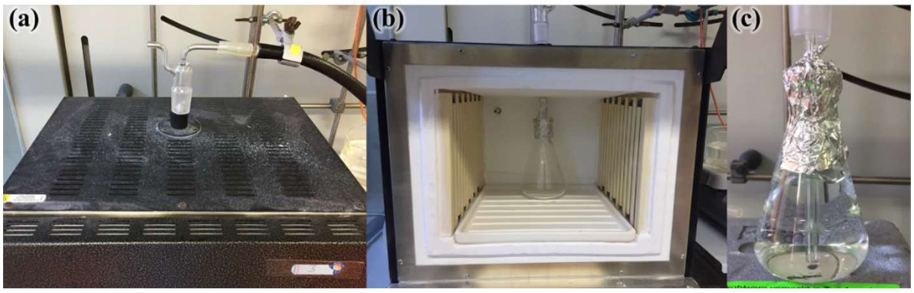

Figure S9. The thermal treatment of the films in nitrogen was performed via a DIY setup. As shown in the above pictures, the films were put in an Erlenmeyer flask with a flow of nitrogen. The flow of nitrogen was quantified according to the bubbles in the water, which was fixed at ~120 bubbles per minute.

Table S1. Photodegradation data of pollutants by different TiO_2-based photocatalysts.

Photocatalyst	Pollutant	State	Light source	Degradation rate to undoped TiO_2 /min^{-1}	Ref.
TiO_2/g-C_3N_4	Cary 60	solution	Halogen lamp 400 − 750 nm	0.023 vs 0.016	S1
TiO_2/Ag/ graphene	methylene blue	solution	Hg lamp 400 – 900 nm	0.000198 vs 0.0000817	S2
N-doped TiO_2/carbon fibers	acid orange 7	solution	Hg lamp 400 – 900 nm	0.0092 vs 0.0011	S3
TiO_2/h-BN	RhB	solution	UV light 365 nm	0.05952 vs 0.01637	S4
	methylene blue			0.0498 vs 0.0118	
TiO_2/ Porous h-BN	reactive yellow 161	solution	Xenon arc lamp 300 – 800 nm	0.01749 vs 0.00444	S5
TiO_2/WS_2	orange II	solution	UV light	0.0480 vs 0.0211	S6
TiO_2/WS_2	methylene blue	solution	Hg lamp	0.03985 vs 0.01407	S7
TiO_2/graphene	stearic acid	solid	UV lamp 365 nm	0.0394 vs 0.0268	S8
TiO_2/h-BN	stearic acid	solid	UV lamp 365 nm	0.0266 vs 0.0175	S9
TiO_2/WS_2	stearic acid	solid	UV lamp 365 nm	0.0121 vs 0.0107	This work
	RhB			0.1102 vs 0.0808	

References

S1. Zhang, Y.; Xu, J.; Mei, J.; Sarina, S.; Wu, Z.; Liao, T.; Yan, C.; Sun, Z., Strongly interfacial-coupled 2D-2D TiO_2/g-C_3N_4 heterostructure for enhanced visible-light induced synthesis and conversion. *J. Hazard. Mater.* **2020**, *394*, 122529. *https://doi.org/10.1016/j.jhazmat.2020.122529*.

S2. Xiao, L.; Youji, L.; Feitai, C.; Peng, X.; Ming, L., Facile synthesis of mesoporous titanium dioxide doped by Ag-coated graphene with enhanced visible-light photocatalytic performance for methylene blue degradation. *RSC Adv.* **2017**, *7*, 25314. *https://doi.org/10.1039/c7ra02198d*.

S3. Li, Y.; Li, M.; Xu, P.; Tang, S.; Liu, C., Efficient photocatalytic degradation of acid orange 7 over N-doped ordered mesoporous titania on carbon fibers under visible-light irradiation based on three synergistic effects. *Appl. Catal. A* **2016**, *524*, 163. *https://doi.org/10.1016/j.apcata.2015.01.050*.

S4. Sheng, Y.; Yang, J.; Wang, F.; Liu, L.; Liu, H.; Yan, C.; Guo, Z., Sol-gel synthesized hexagonal boron nitride/titania nanocomposites with enhanced photocatalytic activity. *Appl. Surf. Sci.* **2019**, *465*, 154. *https://doi.org/10.1016/j.apsusc.2018.09.137*.

S5. Xie, W.; Zhang, M.; Liu, D.; Lei, W.; Sun, L.; Wang, X., Reactive yellow 161 decolorization by TiO_2/porous boron nitride nanosheet composites in cotton dyeing effluent. *ACS Sustainable Chem. Eng.* **2017**, *5*, 1392. *https://doi.org/10.1021/acssuschemeng.6b01896*.

S6. Bassaid, S.; Bellal, B.; Trari, M., Photocatalytic degradation of orange II on the novel hetero-system WS_2/TiO_2 under UV light. *Reac. Kinet. Mech. Cat.* **2015**, *115*, 389. *https://doi.org/10.1007/s11144-015-0845-0*.

S7. Zhang, J.; Zhang, L.; Ma, X.; Ji, Z., A study of constructing heterojunction between two-dimensional transition metal sulfides (MoS_2 and WS_2) and (101), (001) faces of TiO_2. *Appl. Surf. Sci.* **2018**, *430*, 424. *https://doi.org/10.1016/j.apsusc.2017.07.056*.

S8. Malfatti, L.; Falcaro, P.; Pinna, A.; Lasio, B.; Casula, M. F.; Loche, D.; Falqui, A.; Marmiroli, B.; Amenitsch, H.; Sanna, R.; Mariani, A.; Innocenzi, P., Exfoliated graphene into highly ordered mesoporous titania films: highly performing nanocomposites from integrated processing. *ACS Appl. Mater. Interfaces* **2014**, *6*, 795. *https://doi.org/10.1021/am4027407*.

S9. Ren, J.; Stagi, L.; Malfatti, L.; Garroni, S.; Enzo, S.; Innocenzi, P., Boron nitride–titania mesoporous film heterostructures. *Langmuir* **2021**, *37*, 5348. *https://doi.org/10.1021/acs.langmuir.1c00460*.
